# Unveiling Metabolic Phenotype Alterations in Anorexia Nervosa through Metabolomics

**DOI:** 10.3390/nu13124249

**Published:** 2021-11-26

**Authors:** Laura Mayo-Martínez, Francisco J. Rupérez, Gabriel Á. Martos-Moreno, Montserrat Graell, Coral Barbas, Jesús Argente, Antonia García

**Affiliations:** 1 Centre for Metabolomics and Bioanalysis (CEMBIO), Faculty of Pharmacy, Universidad San Pablo-CEU, Montepríncipe Campus, 28660 Madrid, Spain; l.mayo1@usp.ceu.es (L.M.-M.); ruperez@ceu.es (F.J.R.); cbarbas@ceu.es (C.B.); 2 Departments of Pediatrics & Pediatric Endocrinology, Hospital Infantil Universitario Niño Jesús, 28009 Madrid, Spain; gabrielangelmartos@yahoo.es; 3 La Princesa Research Institute, 28006 Madrid, Spain; 4 Department of Pediatrics, Universidad Autónoma de Madrid, 28006 Madrid, Spain; 5 Centro de Investigación Biomédica en Red de Fisiopatología de la Obesidad y Nutriciόn (CIBEROBN), Instituto de Salud Carlos III, 28029 Madrid, Spain; 6 Department of Psychiatry, Hospital Infantil Universitario Niño Jesús, 28009 Madrid, Spain; montserratgraell1@gmail.com; 7 Centro de Investigación Biomédica en Red Salud Mental (CIBERSAM), Instituto de Salud Carlos III, 28029 Madrid, Spain; 8 IMDEA Food Institute, CEI UAM & CSIC, 28049 Madrid, Spain

**Keywords:** anorexia, metabolomics, metabolic phenotype, metabolism, microbiota, mass spectrometry

## Abstract

Anorexia nervosa (AN) is a mental disorder characterized by an intense fear of weight gain that affects mainly young women. It courses with a negative body image leading to altered eating behaviors that have devastating physical, metabolic, and psychological consequences for the patients. Although its origin is postulated to be multifactorial, the etiology of AN remains unknown, and this increases the likelihood of chronification and relapsing. Thus, expanding the available knowledge on the pathophysiology of AN is of enormous interest. Metabolomics is proposed as a powerful tool for the elucidation of disease mechanisms and to provide new insights into the diagnosis, treatment, and prognosis of AN. A review of the literature related to studies of AN patients by employing metabolomic strategies to characterize the main alterations associated with the metabolic phenotype of AN during the last 10 years is described. The most common metabolic alterations are derived from chronic starvation, including amino acid, lipid, and carbohydrate disturbances. Nonetheless, recent findings have shifted the attention to gut-microbiota metabolites as possible factors contributing to AN development, progression, and maintenance. We have identified the areas of ongoing research in AN and propose further perspectives to improve our knowledge and understanding of this disease.

## 1. Introduction

### 1.1. Metabolomics: Basic Concepts and Methodological Aspects

Over the last decades, there has been a shift towards precision and personalized medicine that has led to the development of new ways to approach research in health and disease. New technologies, the so-called “omics”, have emerged to increase the understanding of disease onset and development in a holistic way [1,2].

Metabolomics is the comprehensive analysis of the metabolites included in a specific biological compartment at a specific time (metabolome). The metabolome is highly dynamic and provides valuable information about the ongoing processes in the human body. Since metabolites are the downstream effectors in the molecular pathways (genome—transcriptome—proteome—metabolome), they reflect the changes that have occured at prior stages of these pathways and give an accurate description of the phenotype. In this context, metabolomics has been extensively applied to the discovery of biomarkers for diagnosis, prognosis, and progression of disease in the clinic [3,4].

Importantly, the term metabolomics has been widely applied to studies that cover the metabolic alterations present in different conditions. The most important analytical techniques for the study of the metabolome are nuclear magnetic resonance (NMR) and mass spectrometry (MS). NMR spectroscopy is an analytical technique in which a strong magnetic field is applied to excite the nuclei of the molecular atoms. After that, the atoms return to their lower-energy state, remitting a radiofrequency that is measured by the detector. NMR has been extensively used for the quantitative measurement of metabolites within complex biological matrices [5,6]. MS is an analytical technique based on the formation of ionic species and its later separation according to mass-to-charge ratios under the application of electric or magnetic fields. Additionally, MS-based metabolomics is usually coupled to high-resolution separation techniques: gas chromatography-MS (GC-MS), liquid chromatography-MS (LC-MS), or capillary electrophoresis-MS (CE-MS). It can also be directly performed without metabolite separation by direct infusion (DI) or flow injection analysis (FIA) [7]. Although NMR provides highly accurate and reproducible results in a short time, MS offers higher sensitivity and wider metabolomics coverage, being a powerful platform for metabolomics analyses. Moreover, MS appears to be the optimum technique for targeted approaches, and it has become the most employed technique in metabolomics in recent years [1,8].

According to their scope, metabolomics studies can be generally divided into untargeted and targeted, although sometimes there is an overlap between these approaches [1,9]. Untargeted metabolomics focuses on the global detection and qualitative analysis of all the metabolites present in one sample. These studies are usually performed under discovery stages, where the objective is to gather all the possible information to unveil compounds that could be of interest in a given alteration [1]. Untargeted studies present a complex workflow that comprises analytical procedures but also advanced chemometric analysis to untangle the large amount of information obtained. Due to the wide chemical variability and heterogeneity between the compounds that can be analyzed, multiplatform strategies are employed to broadly cover the metabolome. It is common to combine LC-MS, GC-MS, and CE-MS strategies to analyze all the possible compounds within one single sample, as each technique is best suited for a subset of metabolites with similar physicochemical properties. The main challenge of this first approach is to process all the information that is extracted from each sample, and the main limiting step is the identification and annotation of the compounds found [4,7,10].

On the contrary, targeted metabolomics aims to cover a concrete set of chemically defined metabolites. This is the classical metabolomics approach in which the compounds of interest are previously selected, and then strategies for analysis are defined. The main advantage of targeted metabolomics is that it can be quantitative (absolute concentrations are determined) or semi-quantitative (comparative measurement of metabolite abundances/intensities between groups) [11,12,13], while untargeted metabolomics is a comparative approach, usually between patients and controls. The determining step in targeted metabolomics is to optimize the analytical conditions to enhance the method sensitivity and selectivity to measure the subset of compounds of interest. This approach is usually employed in biomarker validation after a first discovery step, which implies the combination of both metabolomics strategies [3,4].

Metabolomics is therefore a useful methodology to identify novel therapeutic targets and progression or severity biomarkers to develop effective strategies for the treatment or diagnosis of many different diseases, including anorexia nervosa (AN).

### 1.2. Anorexia Nervosa

Anorexia nervosa is a psychiatric disorder characterized by excessive dieting, some compensatory behaviors (excessive exercise, vomiting, and use of laxatives) and, specific psychopathological symptoms (disturbances in the perception of body weight and/or image and fear of becoming fat) that leads to severe and maintained weight loss, which results in progressive malnutrition. The American Psychiatry Association, in the fifth edition of the Diagnostic and Statistical Manual of Mental Disorders (DSM-5) [14], proposed the existence of other restrictive eating disorders such as avoidant/restrictive food intake disorder (ARFID) that presents no specific eating psychopathological symptoms of anorexia (weight concerns and body image disturbance) but features eating or feeding alteration. ARFID includes individuals who meet criteria for the Feeding Disorder of Infancy and Early Childhood DSM-IV category, but also other individuals with clinically significant eating problems who are not included in former DSM categories or therefore must be assigned a diagnosis of eating disorder not otherwise specified (EDNOS), such as selective eating and/or dysphagia [14]. DSM-5 also introduces a new category “Other Specified Feeding or Eating Disorder” (OSFED) for individuals who do not meet criteria for anorexia nervosa, bulimia nervosa or binge disorder and includes five disorders: atypical anorexia, purging disorder, subthreshold bulimia, subthreshold binge eating disorder, and night eating disorder.

AN can be further classified into two main subtypes, restricting and binge/purging disorder. Restrictive anorexia nervosa (AN-R) courses mainly with reduced food intake and excessive exercise, while binge/purging anorexia nervosa (AN-BP) also presents severe energy intake restriction but is combined with recurrent episodes of binge eating or purging behaviors [14,15]. Regardless of the subtype, AN has become one of the most predominant eating disorders with a lifetime prevalence in the general population of 0.6%, being three times higher among females (0.9%) than males (0.3%) [16,17].

The more inclusive DSM-5 criteria reduce the proportion of EDNOS diagnoses regarding DSM-IV and increase the proportion of anorexia and bulimia nervosa, with the new cases probably tending to have a higher minimum body mass index (BMI) and a more benign course [14]. Moreover, it is the eating disorder with the highest mortality rate, mainly due to cardiac complications or suicide [18,19,20].

**Figure 1:**
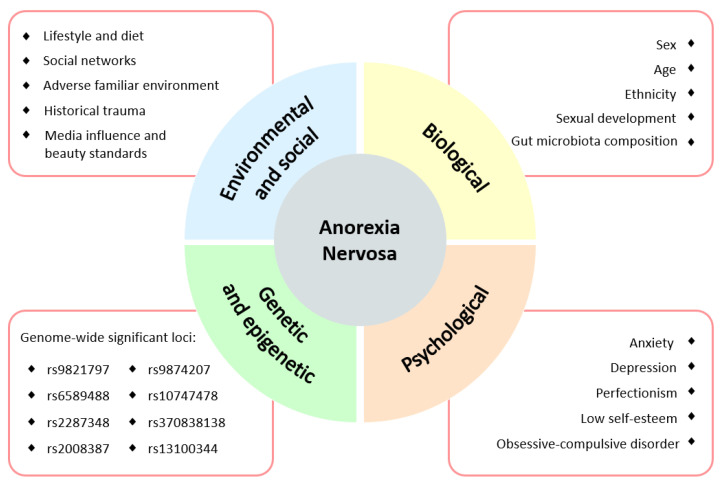
Main factors predisposing to the development of anorexia nervosa [21,22].

Although the etiology of AN remains unclear, there is evidence for disturbed appetite and behavioral pathways that could suggest the physiological origin. Furthermore, it is well established that neurological and genetic predispositions, as well as biological and psychological traits and early experiences in life, might sensitize the individual to stress and hypothalamic-pituitary-adrenal (HPA) axis dysregulation. This sensibility can be further aggravated with environmental and socio-cultural factors that may favor the onset of an eating disorder [21,22]. Thus, AN is postulated to have a multifactorial etiology, and certain conditions may promote the onset in a predisposed population (Figure 1).

Once the disease starts, the maintained weight loss and the altered eating behaviors of the patients lead to wide metabolic dysfunctions complicating the overall clinical picture of the disorder. Among the metabolic alterations, individuals with anorexia nervosa are commonly found to present mild plasma hyper aminoacidemia [23], increased cholesterol levels [24,25], electrolyte imbalances leading to hyponatremia and hypokalemia [26], and profound endocrine disturbances [27,28,29]. AN patients are reported to have lower triiodothyronine (T3) and thyroxine (T4), showing an altered hypothalamic-pituitary-thyroid axis [26]. In addition, increased cortisol levels in serum and urine have been found, suggesting hyperstimulation of the HPA axis [30,31]. Moreover, patients also have distorted appetite-regulating mechanisms, characterized by increased levels of peptide PYY and ghrelin and decreased concentrations of leptin [26,31,32,33,34]. Finally, their extreme eating behaviors lead to micronutrient deficiencies, including reduced levels of zinc, copper, vitamin C, riboflavin, and vitamin B6 [26,35]. Nutritional deprivation can eventually lead to severe complications, including cardiac problems, which is one of the principal causes of death in this disease.

Recent findings in the alteration of intestinal microbiota due to eating behavior and diet have led to increasing interest in microbiota in eating disorders. Few studies have analyzed the microbiota composition in AN, and modifications in microbiome composition and their by-products are scarcely described [18,36,37]. Several fecal metabolites are involved in mood modulation, learning, and memory mechanisms. Regarding this, short-chain fatty acids, lipopolysaccharides from gram-negative bacteria, and neurotransmitters are microbiota metabolites that have autocrine or paracrine functions in the human body [18,38,39]. Moreover, there are chiral metabolites that can have different stereoisomers D and L, with very different biological activity. Amongst these, amino acids and hydroxy acids are involved in neuro-immuno endocrine regulation. The sources of D-amino acids and D-hydroxy acids are food, endogenous enzymatic processes, and the microbiome. The D form of some amino acids, mainly D-serine and D-aspartate, is altered in psychiatric diseases such as schizophrenia or bipolar disorder, but also depression, a common feature in AN [40,41,42,43]. Lactate, a hydroxy acid, is also modified in schizophrenia, depression, and stress disorders [44,45]. Some of these compounds act on receptors in intestinal endothelial cells, and others reach the systemic circulation and can enter the central nervous system, where they mediate different responses [18,38,39]. Thus, the analysis of these microbiota by-products could be relevant for improving our knowledge of the psychopathology of AN. Whether dysbiosis and altered microbiota metabolites are a consequence of malnutrition or if they are involved in AN onset and progression requires further research [21].

To date, the treatment for AN is based on renourishment therapies, as well as psychotherapy and psychopharmacological interventions, to reduce the core psychopathology and the associated disorders (mainly anxiety, depressive and obsessive–compulsive). Unfortunately, specific treatments that target the origin of the disease are still lacking. Thus, there is an increased probability of relapse and to develop a chronic state of the disease, and around 50% of the patients do not achieve full recovery even during a long follow-up period [46]. Treatment objectives should have a strict priority: prevent the death of the patient, prevent the disease from becoming chronic, and attainment of physical and mental recuperation. An integral treatment program should be carried out by a multidisciplinary and coordinated team, including a pediatrician, endocrinologist, psychiatrist, psychologist, and nutritionist.

As a result, the elucidation of the etiology of the disease is of enormous interest to improve treatment and disease outcomes [21]. The pathophysiology of AN could be better elucidated by combining different “omics” approaches to obtain an accurate characterization of the alterations present. In this context, metabolomics appears to be an excellent tool to characterize the metabolic profile of AN patients, leading to the identification of potential alterations that could be useful for the clinical management of anorexia.

The present review aims to present and discuss the information contained in the metabolomics studies that have been performed to date on AN patients with two main objectives: (1) To clearly define the metabolic phenotype of individuals with anorexia nervosa, which is essential for providing new insight into the etiology and pathophysiology of the disease, and (2) To identify the areas that are still uncovered by metabolomics and need further research in the field, with the final purpose of improving disease management and prognosis.

## 2. Background

After a thorough bibliographic query including the terms “Metabolomics” or “Metabonomics” and “Mass Spectrometry” or “Nuclear Magnetic Resonance” and “Anorexia Nervosa”, we found 72 records. Only studies that performed metabolomics (either targeted or untargeted, by NMR or MS) of human samples of AN patients from the last 10 years were included. Thirteen studies were therefore selected for the narrative review and they are summarized in Table 1.

Four studies out of thirteen employed a targeted metabolomics approach: only [47,48,49,50]. Other studies used a combined targeted and untargeted metabolomics approach in their analyses [51,52,53,54]. Most of the studies included used MS for the instrumental analysis. Prochazkova et al. used a combination of NMR and MS for metabolite identification and quantification [54]. Salehi et al. performed serum profiling of AN samples by ^1^H NMR [55].

All the included studies were performed on young adult human females. All the studies performed, with the exception of one, were based on case vs. control analyses, comparing a healthy control group to AN patients. However, ten of them also included patients after treatment, either in the short-term, the long-term, or fully recovered patients [47,48,49,52,53,54,55,56,57,58]. Bulant et al. studied the evolution of the steroid profile of hospitalized women with anorexia nervosa with no control group [50]. One study also performed a timeline analysis including fasting and postprandial samples [56]. Four studies specified that the patients had the restricting type of AN [47,48,51,54]. Moreover, two studies included AN-R and AN-BP patients and established comparisons between them and the healthy controls [37,49]. Three more studies identified both types of AN in their patients, but they did not perform any differential analysis between them [57,58].

Regarding the type of sample analyzed, plasma/serum samples were selected in nine studies [47,48,49,50,51,52,53,55,56], and fecal samples from AN patients were analyzed in the rest of the investigations [37,54,57,58].

## 3. Metabolic Alterations in Anorexia Nervosa

**Figure 2:**
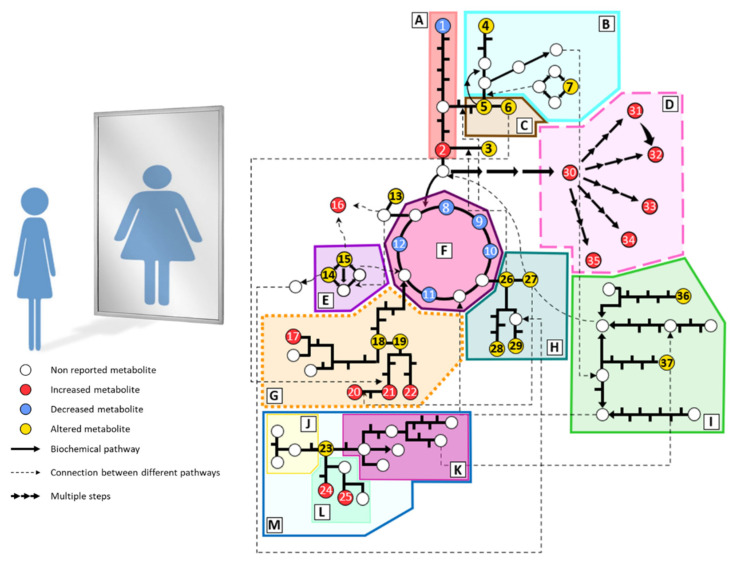
Summary of the main metabolomic alterations found in plasma or serum samples from AN patients in the included studies. Altered pathways: (**A**) glycolysis and gluconeogenesis, (**B**) methionine and cysteine metabolism, (**C**) serine and glycine metabolism, (**D**) lipid metabolism, (**E**) urea cycle, (**F**) tricarboxylate cycle, (**G**) phenylalanine and tyrosine metabolism, (**H**) glutamate, glutamine, proline and histidine metabolism, (**I**) branched-chain amino acids metabolism, (**J**) serotonin pathway, (**K**) kynurenine pathway, (**L**) indole pathway, (**M**) tryptophan metabolism. Metabolites: (1) glucose, (2) pyruvate, (3) alanine, (4) taurine, (5) serine, (6) glycine, (7) methionine, (8) citrate, (9) cis-aconitate, (10) isocitrate, (11) succinate, (12) malate, (13) asparagine, (14) ornithine, (15) arginine, (16) guanidinosuccinate, (17) *p*-cresyl sulfate, (18) tyrosine, (19) phenylalanine, (20) phenylacetylglutamine, (21) phenylacetate, (22) hippurate, (23) tryptophan, (24) indole-3-acetate, (25) indoxyl sulfate, (26) glutamate, (27) glutamine, (28) histidine, (29) proline, (30) fatty acids, (31) phosphatidylcholines, (32) lysophosphatidylcholines, (33) sphingomyelins, (34) acylcarnitines, (35) oxylipins, (36) leucine, (37) isoleucine.

Individuals with anorexia nervosa present severe metabolic disturbances as a consequence of abnormal eating behaviors. Alterations in biochemical parameters have been described in AN (cortisol, cholesterol, electrolytes, etc.). However, the metabolic phenotype or fingerprinting of AN has been scarcely studied. Predominantly, plasma and serum samples are analyzed due to the ease of sample acquisition and the information they provide about the metabolic status. Generally, studies are focused on small groups of metabolites such as amino acids, lipids, or carbohydrates. Hence, wide untargeted metabolomics analyses are still lacking in AN. The main metabolomics alterations found in plasma from AN patients are summarized in Figure 2 and detailed below. Additionally, in Supplementary Table S1 there is a compilation of all the described alterations in human samples of AN found by metabolomics.

### 3.1. Amino Acids

Amino acid dysregulation is usually found in patients with AN, probably related to chronic starvation and altered dietary habits. Some studies have analyzed the amino acid profile in AN patients and the results are inconsistent. However, only a few studies have used metabolomics to assess the amino acidic profile.

#### 3.1.1. Plasma and Serum

M. Föcker et al. performed a **targeted** metabolomics assay to determine 163 metabolites in serum of acute patients, weight-restored patients, and controls. The analysis was done by FIA-MS/MS (Flow injection analysis with tandem mass spectrometry) using the AbsoluteIDQkit^®^ p150 from Biocrates (Innsbruck, Austria). They found mild hyper aminoacidemia in patients, with significantly increased concentrations of glutamine, glycine, histidine, leucine, methionine, ornithine, phenylalanine, serine, and tryptophan [48]. However, in a second study a few years later with the AbsoluteIDQkit^®^ p180, they only reported a significant increase in glutamine, glycine, histidine, serine, and tryptophan [47]. Surprisingly, they described more important metabolic alterations in the weight-restored patients than in the acute phase compared to controls, meaning either that the acute patients adapt to chronic starvation or that the rapid weight gain has a huge impact on metabolism.

Conversely, Miyata et al. studied serum amino acids of individuals with anorexia nervosa through an **untargeted** approach combining UPLC-MS and CE-MS. They found significantly lower values of alanine, asparagine, betaine, histidine, allo-isoleucine, isoleucine, leucine, methionine, proline, taurine, and tyrosine. They also described decreasing tendencies in some other amino acids such as arginine, aspartate, phenylalanine, serine, tryptophan, valine, and threonine. The authors mentioned increasing tendencies in the levels of glutamate, glutamine, glycine, and lysine, although they were not statistically significant. Cysteine levels were not assessed [51].

Burdo et al., in their study on plasma levels of carbon metabolism in AN, reported increased levels of betaine and no variation in methionine in acute patients compared with controls and recovered women [49], contrary to what Miyata et al. and M. Föcker et al. have described [48,51].

Salehi et al. performed a metabolomics study based on ^1^H NMR on serum samples. They compared the profile of acute AN patients (AN) with recovered patients (RecAN) and healthy controls. Five out of twenty-one metabolites were significantly different between the groups. Glutamine was higher in AN when compared to the other groups, but it did not show significant differences between healthy controls and RecAN. Threonine was significantly increased only in RecAN when compared with the AN group. Proline, alanine, serine, and glycine did not show significant variations between the groups [55].

#### 3.1.2. Feces

Monteleone et al. performed an **untargeted** metabolomics assay by GC-MS of fecal samples in acute AN patients, weight restored patients, and healthy controls. They studied 224 identified metabolites, and phenylalanine was significantly decreased in weight-restored patients but acute patients had normal levels compared to healthy controls [57]. However, one year later, Monteleone et al. described decreased levels of phenylalanine, aspartate, serine, and methionine in acute and weight restored AN patients. Leucine was decreased but only in acute patients compared to healthy controls [58]. In 2021, this group performed a new study by using an **untargeted** metabolomics approach with GC-MS in fecal samples comparing both types of AN [37]. They found that isoleucine, leucine, valine, and pyroglutamate are decreased in both types of AN, but the AN-BP subtype presents lower levels than the restricting type compared to controls. Nonetheless, tyrosine and threonine were decreased in AN-R but increased in AN-BP patients.

Although the described amino acid disturbances are not consistent between studies, there is a clear disorder in the amino acidic profile of AN patients. Despite the direction of those variations, we can presume that the mechanisms for homeostasis of amino acids are altered in AN, which in turn leads to modified concentrations of these metabolites in patients. Free amino acid concentrations are the result of the relationship between the incoming sources of amino acid, which include dietary uptake, endogenous synthesis, and gut-bacteria metabolism, and amino acid depletion by protein synthesis and catabolism to increase energy uptake. The altered amino acid pattern is therefore associated with these processes, and whether it is a consequence of chronic starvation or a marked trait of AN that could play a role in the biological origin needs to be clarified.

### 3.2. Lipids

Alterations in the lipid profiles in the plasma, serum, and feces of AN patients have also been described. Distorted eating behaviors, related to fasting and reduction of carbohydrates and fats, produce massive disturbances in metabolism, increasing lipolysis, gluconeogenesis, fatty acid oxidation, and proteolysis [59]. These variations are reflected in the lipidome of individuals with anorexia nervosa. Different strategies have been used to assess the lipid profile in AN.

#### 3.2.1. Plasma and Serum

Föcker et al. studied the serum lipidome by targeted metabolomics. In a first approach, they reported increased lipid concentrations in AN patients during acute starvation and after weight recovery compared to healthy controls. Glycerophospholipids, including phosphatidylcholines (PC), lysophosphatidylcholines (LPC) and sphingomyelins (SM) were significantly increased in patients (e.g., LPC(14:0), LPC(17:0), PC(32:2), PC(32:3), SM(16:0), SM(18:1). In addition, they also observed increased concentrations of some carnitines in serum of AN patients at both time points (e.g., carnitine, acetyl-carnitine) [48]. These results are consistent with previous studies that described the lipid profile of plasma in AN by non-metabolomics approaches [60]. However, Miyata et al. reported that by untargeted metabolomics lower concentrations of some acylcarnitines (AC), such as palmitoylcarnitine, butyrylcarnitine, O-acetylcarnitine, and octanoylcarnitine are observed [51]. Additionally, the changes found by Föcker et al. after renourishment therapy were higher than in the acute state, similar to that found with amino acids, meaning that metabolism is highly susceptible to maintained starvation but even more to the subsequent weight recovery [48]. Nonetheless, in a subsequent study, Föcker et al. found fewer differences between controls and the acute starvation state. The most significant changes were between the starvation state and short-term weight recovery. After complete renourishment therapy, the metabolome was restored, reaching values close to those of the healthy controls. Hence, metabolism seems to adapt to long starvation and renourishment processes, reaching stable metabolic states. The discordances between these studies were justified by the methodological differences and the small sample size in both cases. The most relevant findings in this second study are some compounds that are proposed as potential biomarkers of different states in disease and treatment of AN. These compounds showed significant associations with their respective states and homogeneous time-course behavior in the tested samples. For the starvation state, PC(34:4) and PC(38:3) are significantly decreased and are restored after therapy. In short-term weight recovery, LPC(16:1) and LPC(20:3) are increased, while PC(38:6) and pimelylcarnitine are decreased, suggesting that they could serve as possible markers of the metabolic state during renourishment therapies in AN [47].

Shih et al. used an **untargeted** metabolomics approach by using GC-MS for the determination of polyunsaturated fatty acids (PUFAs) and a **targeted** metabolomics analysis (HPLC-MS/MS) for oxylipins measurement in plasma. Oxylipins are derived from PUFAs by enzymatic (cyclooxygenases, lipoxygenases, and cytochrome P450) or non-enzymatic oxidations, and they are the most relevant mediators of PUFAs functions in the human body. The concentrations of the free fatty acids n-3 (alpha-linolenate-ALA, stearidonate-SDA, eicosapentaenoate-EPA, and docosahexaenoate-DHA) and n-6 (gamma-linolenate-GLA, dihomo-gamma-linolenate-DGLA, arachidonate-ARA, and osbond acid-OBA) were reported to be increased in the plasma of individuals with AN compared to controls. They analyzed the two major ratios between n-3 and n-6 PUFAs (LA (linolenate): ALA and ARA: EPA), which are significantly decreased in AN compared to controls. Moreover, those ratios were inversely correlated with anxiety in individuals with anorexia nervosa, and ARA: EPA was significantly correlated with BMI in patients as well. They also reported significant differences in individual oxylipins and oxylipins ratios. The eicosanoids significantly altered in AN included DHA and ARA metabolites, which belong to the CYP450 pathway. They also studied sEH (soluble epoxide hydrolase) activity which is an enzyme involved in the inactivation of epoxy-fatty acids from CYP catabolism of PUFAs. They finally suggested a greater in vivo activity, concentration, or efficiency of sEH in AN patients when compared to controls. The higher activity of this enzyme, involved in the CYP oxylipin pathway, has been related to increased inflammation and psychiatric disorders such as depression or anxiety, which are comorbidities of AN [61]. As a general overview, individuals with AN showed altered lipidome profiles that were correlated with increased neuroinflammation, anxiety disorders, and lower BMI [52,53].

Nguyen et al. also studied the plasma lipidic profile in acute and recovered individuals with anorexia nervosa compared to healthy controls at two different time points: fasting and postprandial. They examined 26 compounds, including saturated and unsaturated FA by GC-MS. Out of these 26 FA, AN patients presented significant increases in four species under fasting conditions and in only one of them after food intake. Similar to what Shih et al. described, laurate, EPA, and DPA (docosapentaenoate) were increased under fasting while ALA was increased at both timepoints in AN patients [52,56].

Bulant et al. analyzed the steroid profile of serum samples from 33 hospitalized women with AN. The aim was to determine the steroid variations after renourishment therapy. By GC-MS in selected ion monitoring (SIM) mode, they found significantly decreased concentrations of 7β-hydroxy-metabolites of C19Δ5steroids (7β-hydroxydehydroepiandrosterone and 5-androstene-3β,7β,17β-triol) which have been related to immunostimulation and anti-inflammatory properties. They also observed increased concentrations of the steroids at the beginning of the steroidogenic pathway, pregnenolone sulfate, and 20α-dihydro-pregnenolone sulfate after treatment [50].

#### 3.2.2. Feces

Monteleone et al. determined the concentration of some FA in feces by GC-MS. They described lower levels of palmitate in AN-R and AN-BP when compared to healthy controls. Glycerol was also found to decrease in AN-R but not in AN-BP patients. Glycerol depletion can occur as a consequence of starvation due to shifts in the energy sources in carbohydrate deficiency [37]. Monteleone et al. also described increased concentrations of laurate as well as stearic and hydroxystearates in acute patients, but levels were restored after treatment [57].

Overall, the plasma/serum lipidome of individuals with AN is characterized by altered concentrations of n-3 and n-6 FA, glycerophospholipids (PC and LPC), sphingophospholipids (SM), carnitines (AC), steroids; and oxylipins [47,48,50,51,52,53,56]. Additionally, hypercholesterolemia and hyperlipoproteinemia have been widely described in AN patients [24,25,62]. Therefore, LPC, PC, and SM as components of lipoproteins are expected to increase, which is supported by some of the studies mentioned above [47,48]. Moreover, during starvation the lipolysis rate is increased to provide energy substrates for the organism. Hence, triglycerides are hydrolyzed, and FA are mobilized by AC to produce energy through β-oxidation. Therefore, it is plausible that there is an increase of FA and AC in starvation states [48,59,62]. Lipid metabolism is complex and highly variable and can be associated with the state of the disease, sex, age, and more importantly, diet. However, follow-up studies have shown that lipidic profiles are completely restored after treatment, supporting the existence of underlying alterations that need further research [62,63].

### 3.3. Sugars

#### 3.3.1. Plasma and Serum

Carbohydrate profiles also differ in patients and controls. By using ^1^H NMR, Salehi et al. reported lower glucose levels in AN patients compared to controls, but it did not follow the same trend in the recovered patients [55]. In addition, the sum of hexoses determined by Föcker et al. was significantly decreased in acute patients compared to long-term treated patients and controls. In the short-term treated group, hexoses were diminished compared only to healthy controls [47]. In contrast, their previous study showed a higher concentration of hexose in acute and weight-restored patients than in healthy women. However, there were no differences between the patients, either in the acute phase or after treatment [48].

#### 3.3.2. Feces

Monteleone et al. compared the fecal profile of AN-R and AN-BP patients to controls and found that allose, arabinose, lactose, rhamnose, scylloinositol, and xylose were decreased in both groups, but AN-BP presented lower levels when compared to controls. On the contrary, sorbose and tagatose levels were lower in the AN-R group, although both types of patients had significantly decreased concentrations. In summary, a general decrease of carbohydrates was found in the plasma of AN patients independently of their type [37]. Accordingly, in a previous study, they determined that fucose, rhamnose, and xylose were diminished in patients, but normal levels were recovered after renourishment therapy [57,58]. These authors also found that arabinose and tagatose were lower in acute patients, reaching the highest concentration after weight restoration [58].

Altered carbohydrate metabolism is expected in AN. The dietary habits in AN are usually characterized by a low intake of fat and carbohydrate, which makes the organism rely on other sources of energy. Carbohydrate depletion is generally described in undernutrition and starvation. Under fasting conditions, the physiological response involves glycogen breakdown to resort to glucose fuels, which are the main energy source for the cells. Thus, during the early stages of AN, we might find a temporary increase of the carbohydrates in blood that are rapidly consumed. Nevertheless, in chronic starvation, glycogen deposits are exhausted, and there is a shift towards lipolysis and muscle breakdown as energy sources [59].

### 3.4. Tricarboxylate Cycle

Profound metabolic alterations that affect energy metabolism will also impact the tricarboxylate (TCA) cycle. Therefore, disturbances in metabolites within this pathway have been found in AN.

#### 3.4.1. Plasma and Serum

Miyata et al. reported lower levels of intermediates of the TCA cycle in the serum of individuals with AN compared to healthy controls, including malate, succinate, and cis-aconitate [51].

#### 3.4.2. Feces

Likewise, Monteleone et al., in their study of fecal samples from AN patients, reported lower levels of malate in AN-BP and AN-R, while succinate was decreased in AN-R and increased in the AN-BP group [37].

### 3.5. Uremic Toxins

Miyata et al. performed a **targeted** metabolomics analysis of six uremic toxins in serum samples by LC-MS/MS. They found that all, *p*-cresyl sulfate, hippurate, indoxyl sulfate, indole-3-acetate, phenylacetate, and phenyl sulfate, were significantly higher in AN patients versus the control group. Moreover, by an **untargeted** approach, they were able to identify increased concentrations of another two toxins in the AN-R group: guanidinosuccinate and N2-phenylacetylglutamine. Although there was no signal of renal damage in the patients, uremic toxins were increased. As some gut microbiota species can produce uremic toxins, it has been suggested that this increase could be potentially linked to gut dysbiosis in AN patients [51].

### 3.6. Microbial Metabolites

Recent research has focused the attention on the gut-microbiota-brain axis. The impact of gut microbiota on health and disease has recently been described and appears to be an important biological factor in the development and maintenance of EDs. Gut microbiota is defined as the heterogeneous, unique, and dynamic ecosystem of the intestine that depends on complex interactions between genetic and environmental factors [21,64]. Its role in normal physiology and homeostasis is unquestionable. The microbiota is mainly constituted of bacteria, although there are other organisms such as archaea or protozoa. The composition is highly variable among individuals depending on endogenous and exogenous factors such as sex, age, physical activity, genetic features of the host, and infections, among others. However, it has been demonstrated that the predominant factor determining microbiota composition is the diet [36].

The numerous implications of gut microbiota on host health and wellness range from nutrient/energy metabolism to brain function and mood regulation pathways [65]. Moreover, complex direct and indirect interactions between the microbiota, gut, and brain, have been described constituting the termed “microbiota-gut-brain axis”; and microbiota appears to be involved in the regulation of behaviors and emotions, such as learning, stress, depression, and anxiety, that are common traits in AN [39,66].

Once established that the host diet is critical in the gut microbial composition [21] and that patients with EDs have altered nutritional patterns, it can be assumed that these patients will present a modified microbiota [39] and indeed, this has been described in AN. This dysbiosis results from starvation and malnutrition, but the impact on the onset and progression of the disease needs to be further elucidated [18,67,68,69,70,71,72]. Gut microbiota produces a set of bioactive molecules that can induce different responses in the host. Experimental data suggest that an important part of the circulating metabolites in the human body are derived from gut microbiota [73]. Some of these metabolites can interact with receptors in enteroendocrine cells (EECs), and some others can enter systemic circulation performing paracrine functions [74]. Among those metabolites, short-chain fatty acids (SCFAs), neurotransmitters, and lipopolysaccharides are widely studied due to their autocrine and paracrine effects.

To assess the putative effect of dysbiosis on the physiopathology of AN, a combined analytical strategy that determines the composition of the microbiota and its subproducts should be performed. Metabolomics tools can be applied to analyze the products of bacterial metabolism that develop important functions in the human body. To do so, the patient’s fecal sample constitutes a high-value specimen and should be analyzed. However, alterations in feces have been poorly studied for AN.

SCFAs, such as butyrate, propionate, and acetate, are one of the main products of bacterial metabolism. They come from the fermentation of non-digestible carbohydrates, fiber, and resistant starch. SCFAs can target the ENS stimulating the sympathetic nervous system, which is implicated in energy consumption [73,75].

P. Monteleone et al. and A.M. Monteleone et al. performed **untargeted** metabolomics of fecal samples by GC-MS. In their comparative analysis between both anorexia types, they found that acetate was decreased in AN-R patients but not in the AN-BP group [37]. Moreover, they described increased propionate in AN patients that is restored after treatment, contrary to butyrate that is unchanged in patients and decreases after weight recovery [57,58].

Prochazkova et al. performed a multi-omics study with fecal samples from individuals with AN before and after renourishment compared to healthy controls. They determined the composition of gut microbiota and performed **targeted** metabolomics assays for the analysis of fecal SCFAs and neurotransmitters. Butyrate, acetate, and propionate were analyzed by NMR while the neurotransmitters were determined by MS on selective reaction monitoring (SRM). Butyrate was diminished in the ill patients but showed partial recovery after renourishment therapies, although normal values were not achieved. On the contrary, propionate was significantly decreased in patients after treatment, but there were no significant differences in acute patients compared to controls. Acetate levels were significantly lower in both groups of patients, which implies that renourishment does not restore the normal SCFAs profile.

As suggested previously, the changes in fecal metabolites in patients with AN may result from either their chronic malnutrition and/or changes in their gut microbiota composition [37,57]. Regarding this, butyrate has been related to a reduction of anxiety and depressive-like symptoms and to lower neuroinflammation [76,77,78,79]. Thus, decreased butyrate levels might increase susceptibility to depressive-like symptoms. Moreover, the administration of the three SCFAs to mice showed decreased stress-related behaviors [78]. Propionate has also been found to exert direct functions in the central nervous system, it can cross the blood-brain barrier acting on different receptors related to the protection of neuroinflammation mainly [80].

Regarding neurotransmitters, ill patients showed a significant decrease in γ-aminobutyrate (GABA) and dopamine levels. A.M Monteleone et al. also reported significantly decreased GABA levels in both types of AN patients (AN-R and AN-BP) [37]. However, serotonin was only significantly lower in renourished patients. Contrary to expected, the comparison between the patients before and after weight restoration did not yield any significant variation. Tyramine, kynurenine, and hydroxytryptophan concentrations did not vary between groups and they did not change during the course of hospitalization. As a result, novel therapeutic approaches are required to be combined with renourishment to improve the metabolic state of patients [54].

### 3.7. Covariates

A very important issue in clinical trials is the impact of the covariates. In the studies about anorexia discussed in the present review, covariates such as age, BMI, leptin levels are usually recorded and reported, as part of the diagnosis, prognosis and follow-up criteria for the evaluation and management of the disease. Only a few studies included a detailed specific statistical analysis of the impact and association of covariates. Burdo et al. [49] further tested for several associations between nutrient levels and BMI, as well as eating-disorder symptoms, and they reported that vitamin B12 was negatively associated with BMI in AN. Traits of highly specific markers such as the epoxy-fatty acids and other oxylipins were considered together for ANOVA with other covariates such as age, BMI or anxiety [52,53]. Covariates such as BMI and psychiatric comorbidities were also considered [56], but only to evaluate their correlation with the metabolomics findings (individual fatty acids).

## 4. Conclusions and Further Perspectives

AN is a devastating and complex disease with a multifactorial origin. Through targeted and untargeted metabolomics mainly based on 1H NMR or MS, the metabolic phenotype of individuals with anorexia nervosa provided has shed light on the metabolic alterations beyond the classical tests. Alterations in pathways such as glycolysis and gluconeogenesis, methionine and cysteine metabolism, serine and glycine metabolism, lipid metabolism, urea cycle, tricarboxylate cycle, phenylalanine and tyrosine metabolism, glutamate, glutamine, proline and histidine metabolism, branched-chain amino acids metabolism, serotonin pathway, kynurenine pathway, indole pathway, and tryptophan metabolism have been revealed. In particular, gut-microbiota metabolites emerge as possible factors contributing to AN development, progression, and maintenance, and deserve further research.

However, validation is a critical point in untargeted metabolomics studies to avoid chance findings. Discrepancies among different studies can be due to the small sample size and the selection of the control group. For this reason, studies with larger sample sizes considering all possible covariates, and validation of results with targeted analytical methods are compulsory to obtain reliable results. Large cohort studies will diminish chance findings, enforcing the statistical significance of the obtained results. Nonetheless, the recruitment of patients with eating disorders is complicated and hinders the capability of obtaining consistent metabolomic findings.

Moreover, given the intestinal dysbiosis in patients with AN and the two-way communication between the gut microbiota and the brain, known as the “gut-brain axis”, this relationship could be an interesting aim for new studies on the mechanism and development of AN. In this line, putting together information about different samples such as plasma and feces and integrating different omics as well as psychiatric data will give an understanding of this disease and help to design personalized treatments.

In the articles reviewed, sometimes D- or L- amino acids are referred to in the list of metabolites. After careful reading of the full text, no chiral analysis is described, which makes such chiral assignation doubtful and therefore, D- or L- chiral assignation has not been included in this review. Furthermore, up to now, as a global evaluation, chiral assessment in biological samples from patients is scarcely available, and absent in the case of feces samples; thus, it requires further research and development to unveil the role of chirality in anorexia.

Additionally, longitudinal studies have demonstrated that long term starvation has important metabolic consequences in AN patients. Thus, as discussed throughout this review, whether the described metabolic phenotype is a trait marker of the disease or is a consequence of the food deprivation needs to be further elucidated. Importantly, the application of metabolomics approaches could provide more and better information in longitudinal studies about the development of nutritional disorders, such as the Avon Longitudinal Study [81]. Nevertheless, metabolomics in anorexia nervosa has been almost exclusively employed to study single time points, when the disease is already diagnosed, and comparing versus healthy controls.

Notwithstanding the foregoing, although technological advances in the field of omics sciences are making important progress in the application of precision medicine in most medical diseases, further research in the comprehension of the etiology, pathogenesis, and pathophysiology of eating disorders in general and, in the area of AN in particular, are still required. These studies are necessary to facilitate the future successful application of specific targeted therapies for individuals and thus allowing the groundwork for a new era of precision medicine.

## Figures and Tables

**Table 1:** Metabolomics studies on AN in human.

Methodology	Instrumental Analysis	Sample	Study Design	Findings	Ref.
Targeted	FIA-MS/MS	Serum	Evaluation of the metabolic profile of patients during weight recovery. Female adolescents. Healthy controls (*n*= 25) AN patients at inpatient admission (*n* = 35) Short-term weight recovery (*n* = 26) Long-term weight recovery (*n* = 22)	Mild hyper aminoacidemia in patients Increased AC, PC, and SM in patients at different time points Lower sum of hexoses in patients compared to controls FDR	[47]
Targeted	FIA-MS/MS	Serum	Comparison of the metabolic profile of acute patients and short-term weight recovered patients. Young females. Healthy controls (*n* = 16) Acute AN patients (*n* = 29) Short-term weight recovery (*n* = 29)	Mild hyper aminoacidemia in patients Altered lipidic profile: increased AC, LPC and PC, and SM in patients Increased hexoses in patients FDR	[48]
Targeted	LC-MS/MS	Plasma	Analysis of one-carbon metabolism in AN-R and AN-BP patients, in recovered AN patients, and healthy controls. Young women. No eating-disorder history (controls) (*n* = 36) AN-R patients (*n* = 30) AN-BP patients (*n* = 23) AN remitted patients (*n* = 40; 36 with AN-BP history, and 9 with AN-R history)	Increased B12 and betaine in AN active patients compared to controls No differences in choline and Met FDR	[49]
Targeted	GC-MS (SIM)	Serum	Changes in BMI, and psychopathology and steroid metabolome profiling in AN patients before and after hospitalization. Young women. AN patients (*n* = 33)	Increased 20α-dihydro-pregnenolone sulfate and pregnenolone sulfate after treatment 5-androstene-3β,7β,17β-triol, 7β-OH-DHEA, epietiocholanolone, and epipregnanolone were decreased after renourishment	[50]
Untargeted Targeted	UPLC-MS CE-MS LC-MS/MS	Serum	Comparison of the metabolic profile of AN-R patients with age-matching healthy controls. Young females. Healthy controls (*n* = 10) AN-R patients (*n* = 10)	Lower amino acidic levels in patients Decreased AC in patients Lower levels of cis-aconitate, betaine, choline, methyl-2-oxovalerate, and oxovalerate Increased N-phenylacetylglutamine and guanidinosuccinate FDR	[51]
Untargeted Targeted	GC-MS HPLC-MS/MS	Plasma	Multiomics study of AN (genomics, proteomics, and metabolomics). Multiplatform metabolomics study of the lipidome and eicosanoid metabolome of acute AN patients, recovered patients, and healthy controls. Healthy controls (*n*= 36 for PUFAs analysis, *n* = 38 for eicosanoid analysis) Ill AN patients (*n*= 30 for PUFAs analysis, *n* = 10 for eicosanoid analysis) Recovered AN patients (*n*= 30 for PUFAs analysis, *n* = 10 for eicosanoid analysis)	EPHX2 genetic variation is associated with AN development The activity of sEH is elevated in AN compared to controls AN present altered postprandial metabolism of PUFAs and sEH-dependent eicosanoids	[52]
Untargeted Targeted	GC-MS HPLC-MS/MS	Plasma	Evaluation of the lipidomic profile of AN patients compared to healthy controls and recovered patients. Young females. Healthy controls (*n* = 36) Ill AN patients (*n* = 30) Recovered AN patients (*n* = 30)	Increased n-3 and n-6 PUFAs Decreased n-3: n-6 ratios in AN Increased oxylipins from CYP450 pathway	[53]
Untargeted Targeted	^1^H NMR MS (SIM)	Feces	Multiomics approach for analyzing the intestinal microbiota and its metabolites in patients before and after treatment compared to healthy controls. Young females. Healthy controls (*n* = 67) AN-R patients (*n* = 59)	Acetate is decreased before and after treatment Butyrate is decreased in acute patients but increases with renourishment therapy Propionate is lower after treatment and presents no difference in acute patients Decreased dopamine and GABA in acute patients compared to controls Decreased serotonin in recovered patients FDR	[54]
Untargeted	^1^H NMR	Serum	Metabolome profiling of acute AN patients, recovered patients, and healthy controls. Young women. Healthy controls (*n* = 65) AN patients (*n* = 65) Recovered AN patients (*n* = 65)	Gln is higher in acute patients when compared to controls and recovered patients, with no difference between the last two groups Thr is increased only in recovered patients when compared with the acute patients Ala, Gly, Pro, and Ser showed no differences between groups FDR	[55]
Untargeted	GC-MS	Plasma	Evaluation of the fatty acid profile after renourishment therapy in AN patients. Young women. Healthy controls (*n*= 47) Ill AN patients (*n* = 30) Recovered AN patients (*n* = 20)	DPA, EPA, and laurate were increased at fasting compared to controls ALA was increased at both time points compared to controls FDR	[56]
Untargeted	GC-MS	Feces	Comparison of metabolic profiles of patients in an acute state, after recovery, and healthy controls. Young females. Healthy controls (*n* = 20) AN patients (*n* = 24; 18 with AN-R, 6 with AN-BP) AN patients after short-term weight restoration (*n* = 16)	Higher Phe in patients after treatment Higher laurate, hydroxy stearate, and stearate in acute patients Lower fucose, rhamnose, and xylose in acute patients FDR	[57]
Untargeted	GC-MS	Feces	Analysis of the microbiome and the metabolome of AN patients before and after treatment compared to healthy controls. Young females. Healthy controls (*n* = 20) AN patients before treatment (*n* = 21; 16 with AN-R and 5 AN-BP) AN patients after short-term weight restoration (*n* = 16)	Lower Asp, Met, Phe, and Ser in AN patients before and after weight recovery Lower Leu only in acute patients compared to controls Lower fucose, rhamnose, and xylose in patients and their values are restored after treatment Lower arabinose and tagatose in acute patients and increased levels after therapy FDR	[58]
Untargeted	GC-MS	Feces	Evaluation of the microbiome and the metabolic profile of AN-R and AN-BP patients and healthy controls. Young females. Healthy controls (*n* = 20) AN-R patients (*n* = 17) AN-BP patients (*n* = 6)	Lower pyro-Glu, Ile, Leu, and Val in AN-R and AN-BP Lower Thr and Tyr in AN-R Lower palmitate in AN-R and AN-BP Lower glycerol in AN-R Lower allose, arabinose, lactose, rhamnose, scyllo-inositol, sorbose, tagatose, and xylose in AN-R and AN-BP Lower malate in AN-R and AN-BP Lower succinate in AN-R FDR	[37]

## Data Availability

Not applicable.

## Supplementary Materials

The following are available online at https://www.mdpi.com/article/10.3390/nu13124249/s1, Table S1: Metabolites altered in human samples of individuals with AN.

## Abbreviations

^1^H NMR	proton nuclear magnetic resonance
7β-OH-DHEA	7β-hydroxy dehydroepiandrosterone
AC	acylcarnitines
Ala	alanine
ALA	alpha-linolenic acid
AN	anorexia nervosa
AN-BP	anorexia nervosa-binge/purging
AN-R	anorexia nervosa-restricting
Arg	arginine
Asp	aspartate
BMI	body-mass index
CE-MS	capillary electrophoresis-mass spectrometry
DPA	docosapentaenoic acid
EPHX2	epoxide hydrolase 2
EPA	eicosapentaenoic acid
FDR	false discovery rate
FIA-MS/MS	flow injection analysis-tandem mass spectrometry
GABA	γ-amino butyrate
GC-MS	gas chromatography-mass spectrometry
Gln	glutamine
Glu	glutamate
Gly	glycine
HPLC-MS/MS	high performance liquid chromatography-tandem mass spectrometry
Ile	isoleucine
LC-MS	liquid chromatography-mass spectrometry
LC-MS/MS	liquid chromatography-tandem mass spectrometry
Leu	leucine
LPC	lysophosphatidylcholines
Met	methionine
MS	mass spectrometry
NMR	nuclear magnetic resonance
Orn	ornithine
PC	phosphatidylcholines
Phe	phenylalanine
Pro	proline
PUFAs	polyunsaturated fatty acids
sEH	soluble epoxide hydrolase
Ser	serine
SIM	selected ion monitoring
SM	sphingomyelins
Thr	threonine
Trp	tryptophan
Tyr	tyrosine
UPLC-MS	ultra-performance liquid chromatography-mass spectrometry
Val	valine
