# Supplementary material for: Unveiling Metabolic Phenotype Alterations in Anorexia Nervosa through Metabolomics
Source: Nutrients. 2021 Nov 26;13(12):4249. doi: 10.3390/nu13124249 (PMC8706417; doi:10.3390/nu13124249)
Supplement: Supplementary file 1 [file nutrients-13-04249-s001.zip › nutrients-1431258-supplementary.pdf]

nutrients

MDPI

Review

Unveiling Metabolic Phenotype Alterations in Anorexia Nervosa through Metabolomics

Laura Mayo-Martínez, Francisco J. Rupérez, Gabriel Á. Martos-Moreno, Montserrat Graell, Coral Barbas, Jesús Argente, and Antonia García

SUPPLEMENTARY INFORMATION

Supplementary Table S1. Metabolites altered in human samples of individuals with AN.

Metabolite	Sample	Sign of the alteration	Reference
**Glutamine**	Plasma/serum	Altered*	[47,48,51]
**Glycine**	Plasma/serum	Altered*	[47,48,51,55]
**Histidine**	Plasma/serum	Altered*	[47,48,51]
**Leucine**	Plasma/serum	Altered*	[47,48,51]
**Methionine**	Plasma/serum	Altered*	[47–49,51]
**Ornithine**	Serum	Altered*	[47,48]
**Phenylalanine**	Plasma/serum & Feces	Altered*	[47,48,51,57,58]
**Serine**	Plasma/serum & feces	Altered*	[47,48,51,55,58]
**Tryptophan**	Plasma/serum	Altered*	[47,48,51]
**Alanine**	Plasma/serum	Altered*	[51,55]
**Asparagine**	Serum	Altered*	[51]
**Betaine**	Plasma/serum	Altered*	[49,51]
**Allo-isoleucine**	Serum	Decreased	[51]
**Isoleucine**	Serum & feces	Altered*	[37,51]
**Proline**	Plasma/serum	Altered*	[47,48,51,55]
**Taurine**	Serum	Altered*	[51]
**Tyrosine**	Serum & feces	Altered*	[37,51]
**Arginine**	Plasma/serum	Altered*	[47,48,51]
**Aspartate**	Serum & feces	Decreased	[51,58]
**Valine**	Serum & feces	Decreased	[37,51]
**Threonine**	Serum & feces	Altered*	[37,47,48]
**Glutamate**	Serum & feces	Altered*	[51, 37]
**PC**	Plasma	Increased	[47,48]
**LPC**	Plasma	Increased	[47,48]
**SM**	Plasma	Increased	[47,48]
**Carnitines**	Plasma	Altered*	[47,48,51]
**FFA**	Plasma & feces	Increased	[52,53,56,57]
**7β-Hydroxy-dehydro-epi-androsterone**	Plasma	Decreased	[50]
**5-androstene-3β,7β,17β-triol**	Plasma	Decreased	[50]
**Pregnenolone sulfate**	Plasma	Increased	[50]
**20α-dihydro-pregnenolone sulfate**	Plasma	Increased	[50]
**Glycerol**	Feces	Decreased	[37]
**Palmitate**	Feces	Decreased	[37]
**Glucose**	Serum	Decreased	[55]
**Hexoses**	Serum	Altered*	[47,48]

**Allose**	Feces	Decreased	[37,58]
**Arabinose**	Feces	Decreased	[37,58]
**Lactose**	Feces	Decreased	[37]
**Rhamnose**	Feces	Decreased	[37,57,58]
**Scylloinositol**	Feces	Decreased	[37,57,58]
**Xylose**	Feces	Decreased	[37]
**Sorbose**	Feces	Decreased	[37]
**Tagatose**	Feces	Decreased	[37,58]
**Fucose**	Feces	Decreased	[57,58]
**Citrate**	Serum	Decreased	[51]
**Malate**	Serum & feces	Decreased	[37,51]
**Succinate**	Serum & feces	Altered*	[37,51]
**Cis-aconitate**	Serum	Decreased	[51]
**Hippurate**	Serum	Increased	[51]
**P-Cresyl sulfate**	Serum	Increased	[51]
**Indoxyl sulfate**	Serum	Increased	[51]
**Indole-3-acetate**	Serum	Increased	[51]
**Phenylacetate**	Serum	Increased	[51]
**Phenyl sulfate**	Serum	Increased	[51]
**Guanidinosuccinate**	Serum	Increased	[51]
**N2-phenylacetylglutamine**	Serum	Increased	[51]
**Butyrate**	Feces	Decreased	[37,54,57,58]
**Propionate**	Feces	Increased	[37,54,57,58]
**Acetate**	Feces	Decreased	[37,54,57,58]
**GABA**	Feces	Decreased	[37,54]
**Dopamine**	Feces	Decreased	[54]
**Serotonin**	Feces	Decreased	[54]

******Altered* refers to metabolites that have been found significant, but with different variation: increased in some studies, decreased in others.
